# Comparative Phenotypic and Genotypic Characterization of *Salmonella* spp. in Pig Farms and Slaughterhouses in Two Provinces in Northern Thailand

**DOI:** 10.1371/journal.pone.0116581

**Published:** 2015-02-18

**Authors:** Pakpoom Tadee, Phacharaporn Boonkhot, Srirat Pornruangwong, Prapas Patchanee

**Affiliations:** 1 Department of Food Animal Clinic, Faculty of Veterinary Medicine, Chiang Mai University, Chiang Mai, Thailand; 2 Department of Medical Sciences, Ministry of Public Health, Nonthaburi, Thailand; The University of Hong Kong, HONG KONG

## Abstract

*Salmonella* spp. are an important group of bacterial zoonotic pathogens which can cause acute food-borne diseases in humans. Pork products are the main source of salmonellosis, but the origins and transmission routes of the disease have not been clearly determined. The purpose of this study was to characterize *Salmonella* spp. isolated in pig production lines both from pig farms and from slaughterhouses in Chiang Mai and Lamphun provinces in northern Thailand. The study focuses on the association among serotypes, antimicrobial resistance patterns and Pulse Field Gel Electrophoresis (PFGE) patterns to investigate possible sources of infection and to provide information which could help strengthen salmonellosis control programs in the region. A total of 86 strains of *Salmonella* comprising five majority serotypes were identified. Antibiotic resistance to tetracycline was found to be the most prevalent (82.56%) followed by ampicillin (81.40%) and streptomycin (63.95%). Seven clusters and 28 fingerprint-patterns generated by PFGE were identified among strains recovered from various locations and at different times, providing information on associations among the strains as well as evidence of the existence of persistent strains in some areas. Study results suggest that *Salmonella* control programs should be implemented at slaughterhouse production lines, including surveillance to insure good hygiene practices, in addition to regular monitoring of large populations of farm animals.

## Introduction

*Salmonella* has long been associated with food-borne illnesses and is responsible for high rates of morbidity in humans [1–2]. This organism results in significant economic losses and represents a major public health concern worldwide [3–4]. Globally, approximately 90 million cases of gastroenteritis due to *Salmonella* occur annually [5]. There have been many reports of salmonellosis in the northern, central and eastern regions of Thailand [6–8]. The origins and transmission routes of the disease, however, have not been clearly determined.

Pork products are considered to be one important source of *Salmonella* contamination [9–11]. This organism multiplies mainly in the pig’s intestinal tract where it can be detected within two hours of infection [32] However, pigs which are carriers can shed this organism in feces for several weeks or months without showing any clinical signs of infection. *Salmonella*-contaminated pork results from unhygienic slaughtering processes, and infects humans who ingest undercooked product [12]. Curing *Salmonella* infections in humans can be problematic due to the antibiotic resistance of some strains [13] which limits treatment choices and reduces the performance of some first-line treatment options [14].

Bacterial identification is an important part of epidemiological surveillance and outbreak investigation [15]. Serotyping is the most common technique used for *Salmonella* characterization; however, that method is based on immunological typing and thus requires a large number of specific sera [3] and provides a lower discriminatory power than other molecular techniques such as Pulsed Field Gel Electrophoresis (PFGE) [16–18]. PFGE, which is based on gel separation of large DNA fragments generated by digestion with a restriction enzyme [19], is considered the gold standard and the most appropriate method for epidemiological study of the *Salmonella* as it clearly and precisely types several organisms such as *Salmonella* spp., discriminating among related and un-related strains [2]. The purpose of this study was to characterize *Salmonella* spp. isolated from pig production lines both at farms and at slaughterhouses in Chiang Mai and Lamphun provinces in the northern region of Thailand. Focusing on the association of serotypes, antimicrobial resistance patterns and PFGE patterns can help identify possible sources of infection and can provide information to aid the development and implementation of salmonellosis control programs in the region.

## Materials and Methods

### *Salmonella* strains

A total of 86 *Salmonella* strains, *Salmonella* group I 4,5,12:i:- (*n* = 16), *Salmonella* Rissen (*n* = 38), *Salmonella* Stanley (*n* = 16), *Salmonella* Typhimurium (*n* = 10) and *Salmonella* Weltevreden (*n* = 6), were identified during this study (Table 1). Those strains were isolated from pigs, farm and slaughterhouse workers and the local environment around pig farms and pig slaughterhouses in Chiang Mai and Lamphun provinces during the period June 2011 through August 2013.

**Table 1:** Frequency and geographic distribution of *Salmonella* strains isolated from pig farms and pig slaughterhouses in Chiang Mai and Lamphun provinces.

***Salmonella* serotype**	**Area**	**No. of isolates**		
		**farm**	**slaughterhouse**	**Total**
*S*.I. 4,5,12:i:-	Chiang Mai	3	5	8
	Lamphun	7	1	8
*S*.Rissen	Chiang Mai	2	13	15
	Lamphun	12	11	23
S.Stanley	Chiang Mai	0	6	6
	Lamphun	5	5	10
*S*.Typhimurium	Chiang Mai	2	1	3
	Lamphun	6	1	7
*S*.Weltevreden	Chiang Mai	0	3	3
	Lamphun	2	1	3

### Antimicrobial susceptibility testing

All *Salmonella* strains identified were tested for antimicrobial susceptibility by the WHO National *Salmonella* and *Shigella* Center, National Institute of Health, Department of Medical Science, Nonthaburi, Thailand. Each strain was tested with ten different antibiotics using agar disk diffusion [20]. *Escherichia coli* ATCC 25922 was used as the control strain to measure sensitivity. All strains that presented intermediate level resistance were grouped with the susceptible strains to avoid overestimation of resistance. The antibiotics tested included ampicillin (AMP) 10 μg, amoxicillin-clavulanic acid (AUG) 20/10 μg, Chloramphenicol (C) 30 μg, Ciprofloxacin (CIP) 5 μg, Cefotaxime (CTX) 30 μg, Nalidixic acid (NA) 30 μg, Norfloxacin (NOR) 10 μg, Streptomycin (S) 10 μg, Sulfamethoxazole-Trimethoprim (SXT) 23.75/1.25 μg and Tetracycline (TE) 30 μg. Descriptive statistical analysis of the results of these tests was accomplished using Epi Info 7.

### PFGE genotyping

In this study, PFGE genotyping, or genetic fingerprinting, was conducted at the WHO National *Salmonella* and *Shigella* Center, National Institute of Health, Department of Medical Science, Nonthaburi, Thailand, and was accomplished following the US Centers for Disease Control and Prevention (CDC) standardized PulseNet protocol for *Salmonella* [21]. The PulseNet “Universal” standard strain *Salmonella enterica* serovar Braenderup H9812 was used as a reference marker and *Xba*I was used as the digestion enzyme. BioNumerics software version 7.1 was used for cluster analysis of all gel images. Analysis was performed using the unweighted-pair group method, with 2.5% optimization values and 2.5% band position tolerances. Similarity coefficients were acquired using BioNumerics by calculating Dice coefficients. PFGE banding patterns with a similarity index >80% were grouped in the same genotype cluster.

### Discriminatory index

The discriminatory power of PFGE was evaluated using Simpson’s diversity index [22] according to the formula:

$$D=1-\frac{1}{N(N-1)}\sum_{j=1}^{s}nj(nj-1)$$

where *D* is the Simpson’s index of diversity, *N* denotes the total number of strains in the sample population, *S* is the total number of types and n_j_ represents the number of strains belonging to each type. The Simpson’s diversity index estimates the probability that two strains randomly selected from a sample population will belong to the same group. This index calculates values in a range of 0.0 (no diversity) to 1.0 (infinite diversity).

## Results

**Figure 1:**
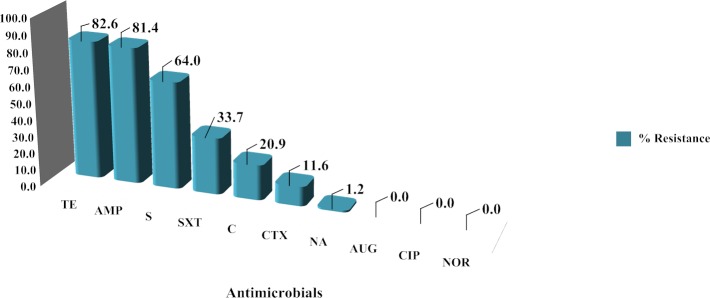
Overview of antibiotic resistance ability of individual *Salmonella* strains. Antibiotic abbreviation: ampicillin (AMP); amoxicillin-clavulanic acid (AUG); chloramphenicol (C); ciprofloxacin (CIP); cefotaxime (CTX); nalidixic acid (NA); norfloxacin (NOR); streptomycin (S); sulfamethoxazole-trimethoprim (SXT); tetracycline (TE).

**Figure 2:**
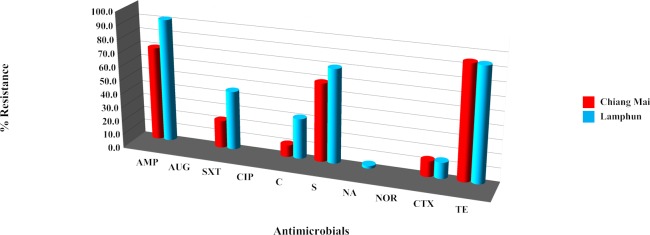
Antibiotic resistance ability of individual *Salmonella* strains by sampling areas. Antibiotic abbreviation: ampicillin (AMP); amoxicillin-clavulanic acid (AUG); chloramphenicol (C); ciprofloxacin (CIP); cefotaxime (CTX); nalidixic acid (NA); norfloxacin (NOR); streptomycin (S); sulfamethoxazole-Trimethoprim (SXT); tetracycline (TE).

**Figure 3:**
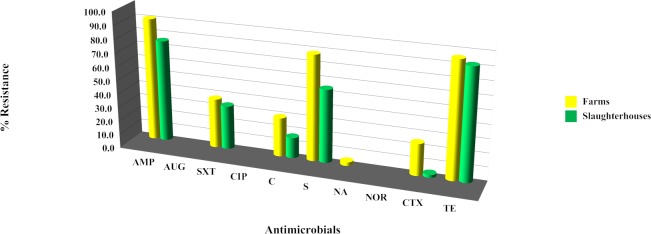
Antibiotic resistance ability of individual *Salmonella* strains by production levels. Antibiotic abbreviation: ampicillin (AMP); amoxicillin-clavulanic acid (AUG); chloramphenicol (C); ciprofloxacin (CIP); cefotaxime (CTX); nalidixic acid (NA); norfloxacin (NOR); streptomycin (S); sulfamethoxazole-Trimethoprim (SXT); tetracycline (TE).

The individual antibiotic resistance profile of each of the 86 *Salmonella* strains was measured. Resistance to tetracycline was the most prevalent among the *Salmonella* strains (71 strains, 82.56%) followed by ampicillin (70 strains, 81.40%) and streptomycin (55 strains, 63.95%) (Fig. 1). Resistance to amoxicillin-clavulanic acid, ciprofloxacin and norfloxacin was not observed in any of the tested strains. Comparison of the antimicrobial resistance ability of *Salmonella* from the two sampling areas showed that resistance to ampicillin, sulfa-trimethoprim, chloramphenicol and streptomycin was greater in samples obtained from Lamphun than in samples from Chiang Mai (Fig. 2). In both areas, the resistance levels of strains from pig farms were higher than strains from slaughterhouses for ampicillin, sulfa-trimethoprim, chloramphenicol, streptomycin, nalidixic acid, cefotaxime and tetracycline (Fig. 3).

**Figure 4:**
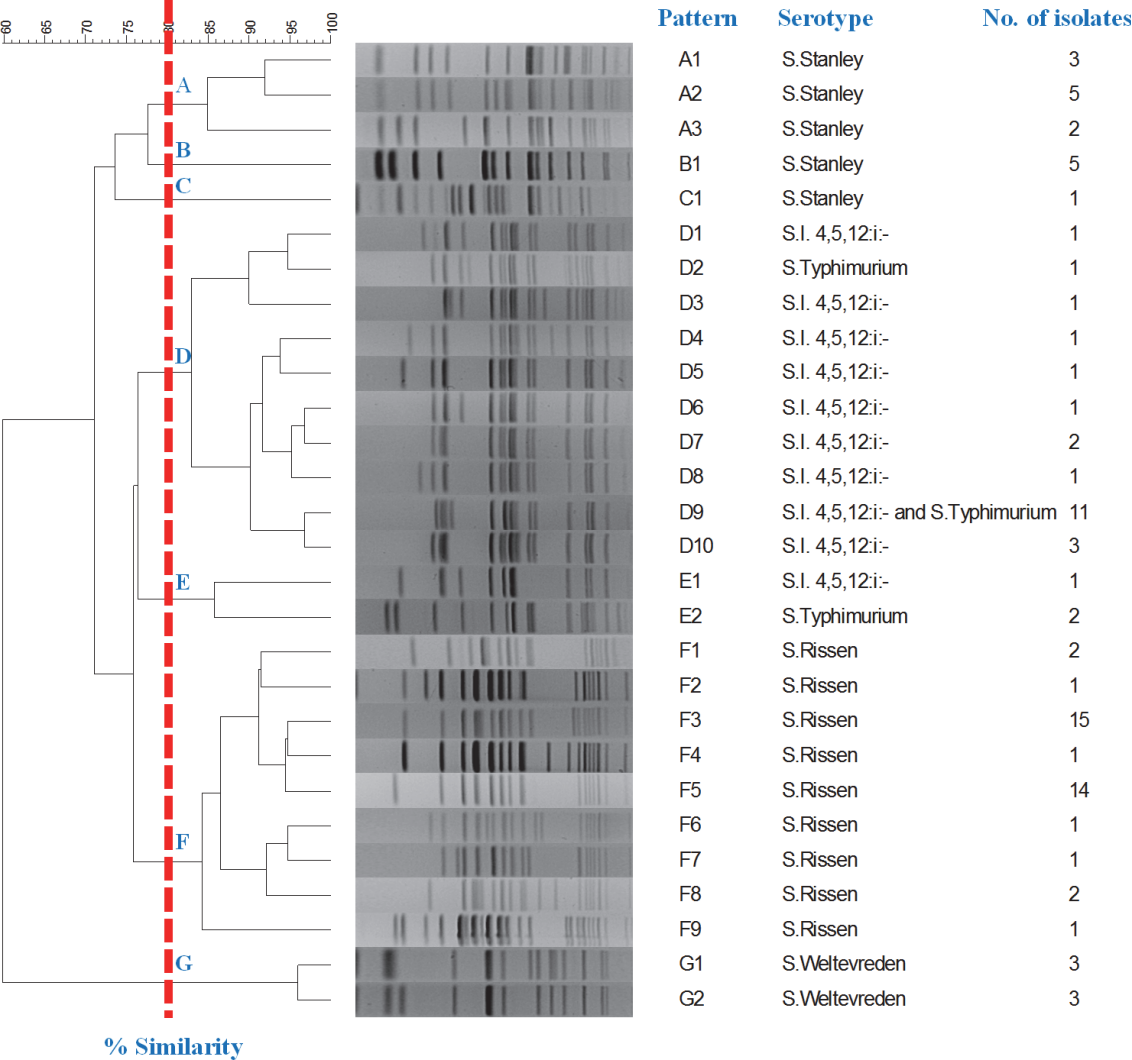
Dendrogram of the 28 patterns PFGE-*Xba*I identified with the frequency of each pattern from five *Salmonella* serotypes isolated from pig farms and pig slaughterhouses in Chiang Mai and Lamphun provinces. (Similarity determined by Dice co-efficient and UPGMA clustering).

Pulse Field Gel Electrophoresis (PFGE) generated profiles of seven major genotypic clusters (A-G) and 28 fingerprint-patterns with an 80% Dice coefficient index cut-off of 12 ∼ 20 DNA fragment bands (Fig. 4). The discriminatory power of Simpson’s diversity index of serotyping and of PFGE were 0.73 and 0.92, respectively. Most strains within a single cluster were of the same serotype, with the exception of *S*.I. 4,5,12:i:- and *S*. Typhimurium which were classified as D and E clusters, respectively. Overall, F-Cluster was the predominant group in this study, comprising 38 strains of *S*. Rissen, followed by D-Cluster (23 strains, 15 from *S*.I. 4,5,12:i:- and 8 from *S*. Typhimurium). All serotypes were divided into groups based on their PFGE patterns. The most common pattern in this study was F3, which included 15 strains of *S*. Rissen, followed by F5 which was composed of 14 strains of *S*. Rissen.

The origins and characteristics of *Salmonella* strains identified in this study are outlined in Table 2. Some indistinguishable strains with diverse origins and phenotypic characteristics were found, especially in the dominant groups (D9, F3 and F5-patterns). Groups of clonal strains isolated from different areas more than 30 km apart and on different days and from different production levels (farm and slaughterhouse) were observed, for example, “SO742/12” and “181/13” in the F3-pattern or “SO895/12” and “37/13” in the F5-pattern. Looking at the strains in more detail, the A1-pattern was found to be composed of three *S*. Stanley, all with similar antimicrobial resistance patterns and all recovered from various processing steps at a single slaughterhouse on the same day. This finding was similar to that for 4 *S*. Rissen in the F3-pattern which was resistant only to tetracycline but also?] to several other antimicrobials with slightly different antimicrobial resistance patterns (4 *S*. Rissen in the F5-pattern collected on 19 May 2013, 2 *S*.I. 4,5,12:i:- in the D7-pattern and 2 *S*. Rissen in the F8-pattern). Groups of indistinguishable strains in samples collected from the same location but on different days were identified from two all-susceptible *S*. Stanley strains isolated from the CN farm in the B1-pattern, three *S*.I. 4,5,12:i:- in the D10-pattern, two *S*. Rissen in the F1-pattern and two *S*. Rissen also obtained from the CN farm in the F3-pattern and six *S*. Rissen in the F5-pattern collected on 19 May 2013, 9 June 2013 and 30 June 2013). Interestingly, the strains with different serotypes were found to be of the D9-pattern which has the greatest diversity in terms of origins and characteristics of 8 *S*.I. 4,5,12:i:- and 15 S. Typhimurium.

**Table 2:** Origin and characterization of *Salmonella* isolated from pig farms and pig slaughterhouses in Chiang Mai and Lamphun provinces.

**Pattern**	**Strains**	**Location**	**Area^a^**	**Source^b^**	**Step**	**Date**	**Serotype**	**ABO- test^c^**
A1	142/13	LPslh	LP	carcass	chilling	7-Jul-13	S. Stanley	AMP,TE
A1	147/13	LPslh	LP	mesen LN	evisceration	7-Jul-13	S. Stanley	AMP,TE
A1	155/13	LPslh	LP	skin	transportation	7-Jul-13	S. Stanley	AMP,TE
A2	SO905/12	SNfarm	LP	feeder	farm	5-Jun-12	S. Stanley	AMP,S,TE
A2	31/13	SPslh	CM	feces	evisceration	26-May-13	S. Stanley	AMP,S,TE
A2	124/13	LPslh	LP	mesen LN	evisceration	9-Jun-13	S. Stanley	AMP,S
A2	125/13	LPslh	LP	hand(aft)	splitting	9-Jun-13	S. Stanley	AMP,S
A2	169/13	BETslh	CM	knife(bef)	cutting&dressing	2-Jun-13	S. Stanley	AMP,S
A3	SO783/12	PDfarm	LP	feces	farm	15-Nov-11	S. Stanley	AMP,S,TE
A3	89/13	SPslh	CM	skin	evisceration	22-Sep-13	S. Stanley	AMP,TE
B1	SO914/12	CNfarm	LP	feces	farm	5-Oct-11	S. Stanley	All Susceptible
B1	SO915/12	CNfarm	LP	floor	farm	12-Jun-12	S. Stanley	All Susceptible
B1	26/13	SPslh	CM	hand(aft)	cutting&dressing	26-May-13	S. Stanley	All Susceptible
B1	29/13	SPslh	CM	hand(aft)	evisceration	26-May-13	S. Stanley	All Susceptible
B1	201/13	BETslh	CM	feces	evisceration	15-Sep-13	S. Stanley	AMP,TE
C1	SO907/12	SNfarm	LP	nipple drinker	farm	5-Jun-12	S. Stanley	AMP,S,TE
D1	172 – BT E22	BETslh	CM	feces	evisceration	2-Jun-13	S.I. 4,5,12: i: -	AMP,S,TE
D2	SO1404/11	DKfarm	CM	feces	farm	23-Jul-11	S. Typhimurium	AMP,C,S,CTX,TE
D3	193—BT D11	BETslh	CM	skin	dehairing	2-Sep-13	S.I. 4,5,12: i: -	AMP,S,TE
D4	A543009	PDfarm	LP	floor	farm	27-Jun-12	S.I. 4,5,12: i: -	AMP,C,S,CTX,TE
D5	A541007	PDfarm	LP	feces	farm	2-Nov-11	S.I. 4,5,12: i: -	AMP,S,CTX,TE
D6	A543008	SNfarm	LP	floor	farm	2-Jun-12	S.I. 4,5,12: i: -	AMP,S,TE
D7	A541024	YPfarm	CM	feces	farm	20-Dec-11	S.I. 4,5,12: i: -	AMP,S,CTX,TE
D7	A541025	YPfarm	CM	feces	farm	20-Dec-11	S.I. 4,5,12: i: -	AMP,C,S,CTX,TE
D8	A541013	PDfarm	LP	feces	farm	15-Nov-11	S.I. 4,5,12: i: -	AMP,S,TE
D9	A543010	YPfarm	CM	floor	farm	3-Jul-12	S.I. 4,5,12: i: -	AMP,SXT,C,S,CTX,TE
D9	60—JRD3	SPslh	CM	knife(aft)	dehairing	4-Aug-13	S.I. 4,5,12: i: -	AMP,S,TE
D9	117—LP L4	LPslh	LP	floor(bef)	lairage	9-Jun-13	S.I. 4,5,12: i: -	AMP,C,S,CTX,TE
D9	173—BT E24	BETslh	CM	mesen LN	evisceration	2-Jun-13	S.I. 4,5,12: i: -	AMP,S,TE
D9	SO1373/11	SNfarm	LP	feces	farm	15-Jun-11	S. Typhimurium	AMP,S,TE
D9	SO1374/11	SNfarm	LP	feces	farm	15-Jun-11	S. Typhimurium	AMP,S,TE
D9	SO1405/11	DKfarm	CM	feces	farm	23-Jul-11	S. Typhimurium	AMP,S,TE
D9	SO1431/11	TDfarm	LP	feces	farm	22-Aug-11	S. Typhimurium	AMP,S,TE
D9	SO1438/11	TDfarm	LP	feces	farm	22-Aug-11	S. Typhimurium	AMP,S,TE
D9	SO1426/11	TDfarm	LP	feces	farm	22-Aug-11	S. Typhimurium	AMP,S,TE
D9	140/13	LPslh	LP	splitter(aft)	splitting	30-Jun-13	S. Typhimurium	AMP,S,TE
D10	A541006	PDfarm	LP	feces	farm	2-Nov-11	S.I. 4,5,12: i: -	AMP,S,CTX,TE
D10	A541011	PDfarm	LP	feces	farm	15-Nov-11	S.I. 4,5,12: i: -	AMP,C,CTX,TE
D10	A541012	PDfarm	LP	feces	farm	15-Nov-11	S.I. 4,5,12: i: -	AMP,S,NA,CTX,TE
E1	21—JRSP3	SPslh	CM	splitter(aft)	splitting	5-May-13	S.I. 4,5,12: i: -	AMP,S,TE
E2	SO1425/11	TDfarm	LP	feces	farm	22-Aug-11	S. Typhimurium	AMP,S,TE
E2	8/13	SPslh	CM	mesen LN	evisceration	5-May-13	S. Typhimurium	AMP,S,TE
F1	40/13	SPslh	CM	skin	evisceration	23-Jul-13	S. Rissen	AMP,S,TE
F1	65/13	SPslh	CM	mesen LN	evisceration	4-Aug-13	S. Rissen	AMP,SXT,S,TE
F2	SO902/12	SNfarm	LP	floor	farm	5-Jun-12	S. Rissen	AMP,SXT,TE
F3	SO742/12	CNfarm	LP	feces	farm	25-Oct-11	S. Rissen	AMP,SXT,C,S,TE
F3	SO762/12	PDfarm	LP	feces	farm	8-Nov-11	S. Rissen	AMP,SXT,C,S
F3	SO921/12	CNfarm	LP	floor	farm	12-Jun-12	S. Rissen	All Susceptible
F3	SO1402/11	SNfarm	LP	floor	farm	5-Jun-12	S. Rissen	AMP,SXT,TE
F3	SO1429/11	TDfarm	LP	feces	farm	22-Aug-11	S. Rissen	AMP,SXT,C,S,TE
F3	SO1430/11	TDfarm	LP	feces	farm	22-Aug-11	S. Rissen	AMP,SXT,C,S,TE
F3	28/13	SPslh	CM	mesen LN	evisceration	26-May-13	S. Rissen	AMP,S,TE
F3	34/13	SPslh	CM	carcass	splitting	26-May-13	S. Rissen	AMP,SXT,S,TE
F3	100/13	LPslh	LP	table(bef)	cutting&dressing	19-May-13	S. Rissen	AMP,SXT,TE
F3	101/13	LPslh	LP	hand(aft)	cutting&dressing	19-May-13	S. Rissen	AMP,SXT,C,S,TE
F3	114/13	LPslh	LP	carcass	splitting	19-May-13	S. Rissen	AMP,SXT,C,S,TE
F3	178/13	BETslh	CM	knife(aft)	bleeding	23-Jun-13	S. Rissen	TE
F3	181/13	BETslh	CM	knife(aft)	dehairing	23-Jun-13	S. Rissen	TE
F3	182/13	BETslh	CM	feces	evisceration	23-Jun-13	S. Rissen	TE
F3	187/13	BETslh	CM	carcass	splitting	23-Jun-13	S. Rissen	TE
F4	SO926/12	YPfarm	CM	feed	farm	3-Jul-12	S. Rissen	AMP,SXT,S,TE
F5	SO790/12	PDfarm	LP	feces	farm	22-Nov-11	S. Rissen	AMP,SXT,TE
F5	SO895/12	TDfarm	LP	floor	farm	5-Sep-11	S. Rissen	AMP,SXT,S,TE
F5	SO898/12	DKfarm	CM	boot	farm	24-May-12	S. Rissen	AMP,SXT,S,TE
F5	SO1386/11	SNfarm	LP	feces	farm	15-Jun-11	S. Rissen	AMP,SXT,S,TE
F5	SO1403/11	SNfarm	LP	floor	farm	5-Jun-12	S. Rissen	AMP,SXT,TE
F5	14/13	SPslh	CM	hand(aft)	cutting&dressing	26-May-13	S. Rissen	TE
F5	25/13	SPslh	CM	knife(aft)	dehairing	26-May-13	S. Rissen	TE
F5	37/13	SPslh	CM	truck	transportation	26-May-13	S. Rissen	AMP,SXT,TE
F5	105/13	LPslh	LP	carcass	chilling	19-May-13	S. Rissen	AMP,SXT,C,S,TE
F5	107/13	LPslh	LP	hand(aft)	splitting	19-May-13	S. Rissen	AMP,SXT,S,TE
F5	109/13	LPslh	LP	floor(bef)	lairage	19-May-13	S. Rissen	AMP,SXT,S,TE
F5	115/13	LPslh	LP	splitter(aft)	splitting	19-May-13	S. Rissen	AMP,SXT,C,S,TE
F5	126/13	LPslh	LP	carcass	washing	9-Jun-13	S. Rissen	AMP,SXT,S,TE
F5	139/13	LPslh	LP	floor(aft)	lairage	30-Jun-13	S. Rissen	AMP,SXT,TE
F6	69/13	SPslh	CM	truck	transportation	4-Aug-13	S. Rissen	AMP,SXT,S,TE
F7	45/13	SPslh	CM	cage	transportation	23-Jul-13	S. Rissen	AMP,TE
F8	132/13	LPslh	LP	knife(aft)	dehairing	30-Jun-13	S. Rissen	AMP,SXT,C,TE
F8	134/13	LPslh	LP	feces	evisceration	30-Jun-13	S. Rissen	AMP,SXT,C,S,TE
F9	SO741/12	CNfarm	LP	feces	farm	25-Oct-11	S. Rissen	AMP,SXT,C,S,TE
G1	SO922/12	CNfarm	LP	nipple drinker	farm	12-Jun-12	S. Weltevreden	All Susceptible
G1	44/13	SPslh	CM	carcass	chilling	23-Jul-13	S. Weltevreden	All Susceptible
G1	204/13	BETslh	CM	feces	evisceration	15-Sep-13	S. Weltevreden	All Susceptible
G2	SO923/12	CNfarm	LP	nipple drinker	farm	12-Jun-12	S. Weltevreden	AMP,C,S
G2	30/13	SPslh	CM	feces	evisceration	26-May-13	S. Weltevreden	All Susceptible
G2	122/13	LPslh	LP	feces	evisceration	9-Jun-13	S. Weltevreden	All Susceptible

^a^Abbreviations in this column: mesenteric lymphnode (mesen LN); before operation (bef); after operation (aft).

^b^Abbreviations in this column: Chiang Mai province (CM); Lamphun province (LP).

^c^Antibiotic abbreviations: ampicillin (AMP); amoxicillin-clavulanic acid, (AUG); Chloramphenicol (C); Ciprofloxacin (CIP); Cefotaxime (CTX); Nalidixic acid (NA); Norfloxacin (NOR); Streptomycin (S); Sulfamethoxazole-Trimethoprim (SXT); Tetracycline (TE).

## Discussion

Of the 86 *Salmonella* strains tested, most were resistant to at least one antimicrobial agent. Tetracycline, ampicillin, and streptomycin were found to have a higher resistance rate than other antimicrobial agents. This finding is similar to studies conducted in Ireland [23], Belgium [14] and Germany [24]. Based on those findings, the betalactam, aminoglycoside and tetracycline groups are not recommended for salmonellosis treatment. The excessive or inappropriate use of those antimicrobial agents in livestock, either as a treatment of disease or as a prophylactic, is considered to be a key factor leading to the current resistance situation [25–27]. However, an absence of resistance to amoxicillin-clavulanic acid, norfloxacin and ciprofloxacin was observed, a finding similar to a study in Sa Kaeo Province, Thailand [8]. The lack of resistance observed might be due to the limited use of those specific antimicrobial drugs in pig production in Thailand. In this study, almost all resistance rates in *Salmonella* obtained from Chiang Mai were lower than those from Lamphun, even though the opportunity to obtain accurate information on antimicrobial use by farm owners was greater in Chiang Mai than in Lamphun. All *Salmonella* resistance rates in samples obtained from farms were higher than those obtained from slaughterhouses in both provinces. That finding contrasts with the observation by Schwaiger et al. [24] and Mc Mahon et al. [27] that meat is a particularly suitable matrix for bacteria. Stress factors such as unsuitable temperatures or pH levels as well as other sub-lethal stress-producing conditions in various slaughtering steps could play a role in enhancing antimicrobial resistance. The reason for this study’s finding of higher resistance on farms than at slaughterhouses was not immediately obvious.

Comparison of the discriminatory power of serotyping with PFGE using Simpson’s diversity index found that the serotyping method had only a weak ability to differentiate between related and un-related strains (*D* = 0.73) compared with the PFGE method (*D* = 0.92). This result is consistent with a PFGE study of 190 *S*. enterica by Soyer et al. [28] which reported a PFGE of *D* = 0.96 and a study conducted in 128 *S*. Enteritidis by Campioni et al. [2] which found a PFGE of *D* = 0.98, indicating that PFGE is the most appropriate technique for *Salmonella* typing [29].

Following standard sampling methodology, the five serotypes selected in this study were among those commonly found in northern Thailand. Representative strains of each serotype were randomly selected using stratified sampling. Thus *S*. Rissen was the major serotype in this study. *S*. Rissen has also been reported to be the dominant serotype found in pig production lines in this region for the last eight years [6, 30].

Twenty-eight unique PFGE patterns were generated. Most of the patterns were correlated with one serotype; however, some patterns did not match well. Four and seven serotypes of *S*.I. 4,5,12:i:- and *S*. Typhimurium, respectively, were found to be in the D9-pattern. Because *S*. Typhimurium has the antigenic formula 4,5,12:i:1,2, a possible explanation is that the serotype evolved from a common ancestor with *S*.I. 4,5,12:i:- but that present second-phase of flagella antigens [28]. The same reasoning could also explain the arrangement position of PFGE and the genetically similar profiles (the antigenic formula of *S*. Stanley is 4,5,12:d:1,2 and of *S*. Weltevreden is 3,10:r:z_6_ [31]).

The indistinguishable strains were obtained from various areas and at different sampling times which indicates that those strains probably have some association. Most slaughterhouses in northern Thailand receive finisher pigs only from nearly areas; receipt of finisher pigs from another province would be very unlikely. That indicates that cross-contamination might start at the farm level: *Salmonella* may spread over a wide area via the supply chain (e.g., gilts, feed, feed-ingredients) although finisher pigs are considered the main source of contamination along the farm to slaughterhouse route.

The identical PFGE patterns in strains recovered from various production steps in a single area during one sampling day indicate cross contamination within those areas. *Salmonella* carriers in an area might have shed bacteria which were then transferred to *Salmonella* free-pigs directly or via the environment. Moreover, inadequacies in routine production practices also promote the colonization and spread of *Salmonella* to pork via contaminated carcasses, slaughtering equipment or worker’s hands at any of the slaughtering-steps [24, 33, 34]. Additionally, the diversity of some phenotypic characteristics such as antimicrobial resistance patterns might increase by means of recombination, mutation or horizontal gene transfer [3]. Furthermore, the fact those groups of indistinguishable strains were isolated from the same location on different days is evidence of the persistence of some strains [23]. This idea is given credence by the detection of “109/13” in the F5-pattern in samples recovered from the lairage floor before use of that facility and then again two days later, after it had been used. This indicates improper cleaning or inadequate hygienic practices in the lairage area of this slaughterhouse.

## Conclusions

The results demonstrate that PFGE delivers more discriminatory power for *Salmonella* identification and that the technique can provide valuable information for disease surveillance and outbreak investigation. They also highlight the emergence of persistent strains and the association of clonal strains recovered from various areas at both the pre-harvest and post-harvest levels. Additional observations will be needed to further identify the links to strains recovered at the next production level. Nevertheless, there is a need to conduct control programs to improve biosecurity and hygienic practices at individual localities as well as along the entire production line. The diverse phenotypic characteristics such as antibiotic resistance patterns demonstrated among clonal strains might be caused by recombination, mutation or horizontal gene transfer. To confirm this additional hypothesis, a resistance gene study should be performed.

## Supporting Information

S1 Table Names and locations of target pig farms and slaughterhouses in this study. Accession to farms and slaughterhouses were permitted by livestock standard and certification unit under Livestock administrative region 5 (8 provinces in Northern Thailand). Dr. Chairoj Pocharoen, DVM (chairojp@hotmail.com), contracting governmental officer, was a person responsible for farm and slaughterhouse sample collections. All fecal samples were collected from rectum of pigs by finger palpation method from farm (data available at http://www.ncbi.nlm.nih.gov/pmc/articles/PMC4087236/) whereas fecal samples samples and mesenteric lymhnode samples from slaughterhouses were obtained directly from rectum and intestines, respectively, over the evisceration step. The remaining of cotton swab samples were collected from several farm environments and slaughterhouse facilities.
(DOCX)
