# Supplementary material for: Comparative Phenotypic and Genotypic Characterization of Salmonella spp. in Pig Farms and Slaughterhouses in Two Provinces in Northern Thailand
Source: PLoS One. 2015 Feb 18;10(2):e0116581. doi: 10.1371/journal.pone.0116581 (PMC4334970; doi:10.1371/journal.pone.0116581)
Supplement: S1 Table — Accession to farms and slaughterhouses were permitted by livestock standard and certification unit under Livestock administrative region 5 (8 provinces in Northern Thailand). Dr. Chairoj Pocharoen, DVM (chairojp@hotmail.com), contracting governmental officer, was a person responsible for farm and slaughterhouse sample collections. All fecal samples were collected from rectum of pigs by finger palpation method from farm (data available at http://www.ncbi.nlm.nih.gov/pmc/articles/PMC4087236/) whereas fecal samples samples and mesenteric lymhnode samples from slaughterhouses were obtained directly from rectum and intestines, respectively, over the evisceration step. The remaining of cotton swab samples were collected from several farm environments and slaughterhouse facilities. (DOCX) [file pone.0116581.s001.docx]

**Table S1. Names and locations of target pig farms and slaughterhouses in this study**

| **Production levels** | **Names** | | **Locations** |
| --- | --- | --- | --- |
| **Farms** | | Donkaew farm | Mae Rim district, Chiang Mai province |
|  | | Yupa farm | Doi Saket district, Chiang Mai province |
|  | | Padet farm | Meaung district, Lamphun province |
|  | | Teerada farm | Pa Sang district, Lamphun province |
|  | | Sanan farm | Meaung district, Lamphun province |
|  | | Chamnan farm | Meaung district, Lamphun province |
|  | |  |  |
| **Slaughterhouses** | | Chitrungreuang slaughterhouse | San Kampang district, Chiang Mai province |
|  | | Lamphun co-operative slaughterhouse | Meaung district, Lamphun province |
|  | | Betagro slaughterhouse | Mae Rim district, Chiang Mai province |

Accession to farms and slaughterhouses were permitted by livestock standard and certification unit under Livestock administrative region 5 (8 provinces in Northern Thailand). Dr. Chairoj Pocharoen, DVM ([chairojp@hotmail.com](mailto:chairojp@hotmail.com)), contracting governmental officer, was a person responsible for farm and slaughterhouse sample collections. All fecal samples were collected from rectum of pigs by finger palpation method from farm (data available at http://www.ncbi.nlm.nih.gov/pmc/articles/PMC4087236/) whereas fecal samples samples and mesenteric lymhnode samples from slaughterhouses were obtained directly from rectum and intestines, respectively, over the evisceration step. The remaining of cotton swab samples were collected from several farm environments and slaughterhouse facilities.
